# Qualitative and quantitative analyses of polysomnographic measurements in foals

**DOI:** 10.1038/s41598-021-95770-5

**Published:** 2021-08-11

**Authors:** Antonia Zanker, Anna-Caroline Wöhr, Sven Reese, Michael Erhard

**Affiliations:** 1 Tierärztliche Klinik Für Pferde Wolfesing, Wolfesing 12, 85604 Zorneding, Germany; 2 Chair of Animal Welfare, Animal Behaviour, Animal Hygiene and Animal Husbandry, Department of Veterinary Sciences, Faculty of Veterinary Medicine, LMU Munich, Veterinärstraße 13 R, 80539 München, Germany; 3 Chair of Anatomy, Histology and Embryology, Department of Veterinary Sciences, Faculty of Veterinary Medicine, LMU Munich, Veterinärstraße 13 R, 80539 München, Germany

**Keywords:** Physiology, Zoology, Health care, Medical research, Neurology

## Abstract

Veterinary and human medicine are still seeking a conclusive explanation of the function of sleep, including the change in sleep behaviour over the course of an individual’s lifetime. In human medicine, sleep disorders and abnormalities in the electroencephalogram are used for prognostic statements, therapeutic means and diagnoses. To facilitate such use in foal medicine, we monitored 10 foals polysomnographically for 48 h. Via 10 attached cup electrodes, brain waves were recorded by electroencephalography, eye movements by electrooculography and muscle activity by electromyography. Wireless polysomnographs allowed us to measure the foals in their home stables. In addition, each foal was simultaneously monitored with infrared video cameras. By combining the recorded data, we determined the time budgeting of the foals over 48 h, whereby the states of vigilance were divided into wakefulness, light sleep, slow-wave sleep and rapid-eye-movement sleep, and the body positions into standing, suckling, sternal recumbency and lateral recumbency. The results of the qualitative analyses showed that the brain waves of the foals differ in their morphology from those previously reported for adult horses. The quantitative data analyses revealed that foals suckle throughout all periods of the day, including night-time. The results of our combined measurements allow optimizing the daily schedule of the foals according to their sleep and activity times. We recommend that stall rest should begin no later than 9.00 p.m. and daily stable work should be done in the late afternoon.

## Introduction

In human medicine, it is now possible to use polysomnographic data from new-borns to make statements about further brain development^1^. Thus, a prognostic assessment of the viability of the new-born is possible by analysing the polysomnographic data. Besides such prognostic uses, polysomnographic data of human new-borns have been used for the recognition and diagnosis of sleep apnoea^2^ and epileptic fits^3,4^. Moreover, the neurophysiological mechanisms associated with sleep and wakefulness in humans are well-researched^5^, and much of this knowledge has been applied in veterinary research.

Polysomnographic studies on horses have clearly advanced over the last 20 years. Cable-free equipment and non-invasive electrodes now allow polysomnographic examinations in the horses’ stables^6–8^. In addition, normative data for various states of vigilance in adult horses have been established by using electroencephalogram (EEG), electrooculogram (EOG) and electromyogram (EMG) recordings^8,9^. Polysomnography on adult horses has been used to support behavioural research^9–13^, to explore the impact of sedatives and narcotics^14–21^ and to diagnose diseases^22–25^.

Polysomnographic studies on foals included research on sedatives^16^, on diseases^26–28^, and on the attachment of electrodes^6^. In various observational studies, the lying behaviour of young foals was recorded^29–32^. However, a foal in a lying position might be awake or asleep, and the recording of lying behaviour alone does not allow differentiation between states of vigilance such as wakefulness (i.e. being awake as opposed to being asleep), light sleep, slow-wave sleep or rapid-eye-movement (REM) sleep^8,9^. To our knowledge, neither polysomnographic measurements of healthy foals over several nights and days nor studies on sleep phases of foals in general or in combination with the body position have been published.

The aim of this study was therefore to simultaneously measure the sleep and lying behaviour of healthy foals in their familiar environment. Using polysomnographic data and synchronized video recordings over 48 h, we determined EEG values as well as daytimes and average durations for different states of vigilance in foals. Based on the results, we were able to classify the different vigilance stages based on frequency ranges in Hertz (Hz) and assign grapho-elements (e.g. spindles) to these stages. In addition, we present a foal-suitable method of electrode application and discuss the benefits of combining polysomnographic measurements with synchronized video recordings for qualitative and quantitative data analyses. The results will serve as basis for further studies on drug effects, early detection of diseases and diagnosis of brain maldevelopment and can help derive recommendations for animal-friendly keeping of foals. Furthermore, the results may stimulate research on using brain wave patterns to determine the survivability of so-called dummy foals—on the one hand to spare the animals avoidable pain, suffering and damage, and on the other hand to improve economic aspects of foal husbandry.

## Results

### Sleep behaviour

The polysomnographic EEG frequencies of adult horses cannot be used in foals because of the different appearance of the EEG waves. We noticed that δ-waves dominated in all vigilance states of the foals. The exact description of the EEGs and the proposed guideline values will be presented and discussed per individual vigilance stage.

**Figure 1:**
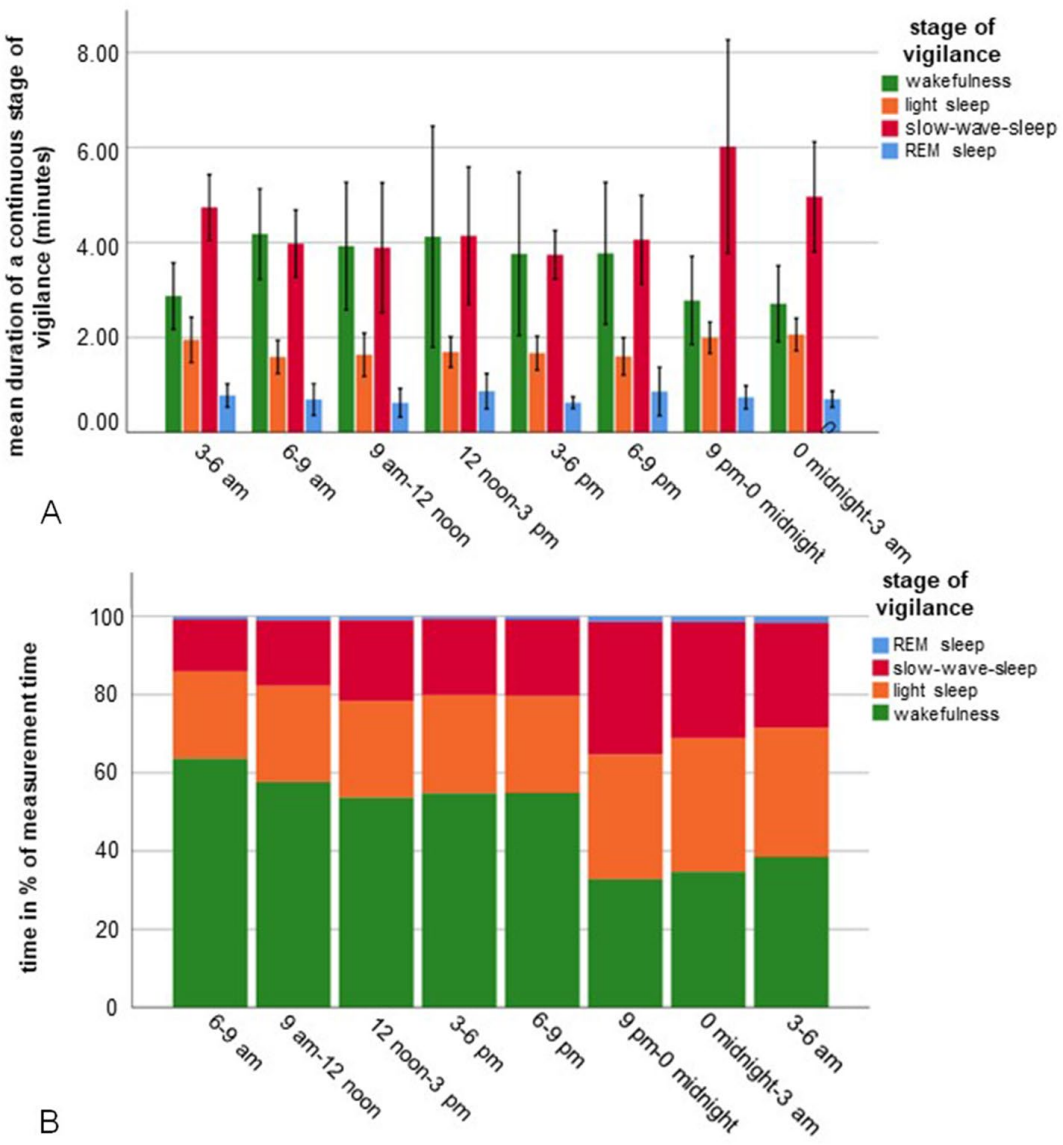
Duration (**A**) and percentage (**B**) of the four stages of vigilance in foals (n = 10) during the three-hour periods of a one-day cycle (total measurement time 48 h). The x-axis shows the three-hour periods of the 24-h cycle. The y-axis shows the sequence duration in minutes (**A**) or the percentage (**B**) of each stage of vigilance. A sequence represents a single continuous vigilance stage. The stages of vigilance are shown in different colours (green: wakefulness; orange: light sleep; red: slow-wave sleep; blue: REM sleep).

Using the polysomnographic data in combination with the video recordings, we assigned four vigilance stages. These were wakefulness, light sleep, slow-wave sleep and REM sleep. The percentage of the total measurement time, the duration of each sequence and the relationship between sleep stage and body position are shown in Table 1 and Fig. 1A,B.

**Table 1:** Overview of the percentage of the total measurement time (48 h) and the duration of the four stages of vigilance in foals (n = 10) and the percentage of each vigilance stage spent in the four body positions.

Parameter	Stage			
	Wakefulness	Light sleep	Slow-wave sleep	REM sleep
% of measurement time	48.8	27.6	22.5	1.1
Average duration (± standard deviation) of a sequence^1^ in minutes	3.3 (± 4.4) Minimum 0.5 Maximum 76.0	1.8 (± 1.8) Minimum 0.5 Maximum 23.0	4.4 (± 3.9) Minimum 0.5 Maximum 30.0	0.8 (± 0.5) Minimum 0.5 Maximum 3.5
% in standing position	36.1	4.6	3.8	0
% during suckling	26.2	32.7	0	0
% in sternal recumbency	23.2	26.2	36.0	22.3
% in lateral recumbency	14.4	36.6	60.1	77.7

^1^Sequence = A single continuous vigilance stage.

### Wakefulness

During wakefulness, the foals were standing, moving around or lying down while actively engaging with their environment. Muscle- and movement artefacts superimposed the waves of the EEG. There was no clear basic brain wave activity such as an α-rhythm. Depending on the type of activity, the EOG showed slow to fast eye movements. The EMG during wakefulness was characterized by the highest frequency and highest amplitude as compared with the sleep stages. Depending on the activity levels of the foals, the EEG waves showed a broad frequency spectrum with varying frequency ranges and varying amplitudes. On average, wakefulness was most prevalent (63.6% of the measurement time) between 6.00 and 9.00 a.m. and least prevalent (32.8% of the measurement time) between 9.00 p.m. and midnight (Fig. 1B). The average duration of a waking sequence was 3.79 min (standard deviation ± 0.84; minimum 0.5 min; maximum 36.5 min) during the day and 2.73 min (± 0.59; minimum 0.5 min; maximum 41.5 min) during the night (Fig. 1A). Throughout the day, the foals spent more time awake than during the night (t-Test t = 10.093, *p* < 0.001). No differences could be observed in the total percentage of wakefulness between the three-hour periods of the day (ANOVA, F = 1.532, *p* = 0.209) or between those of the night (ANOVA, F = 2.192, *p* = 0.131).

### Light sleep

**Figure 2:**
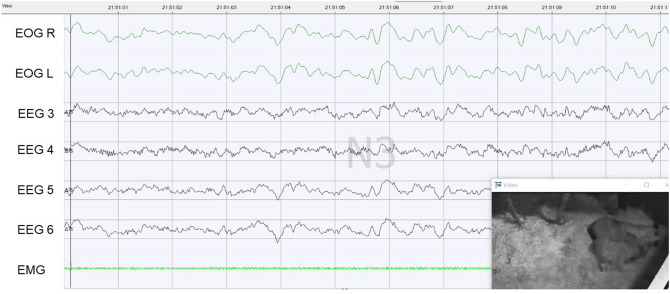
Light sleep of a foal lying in lateral recumbency. The amplitudes are within the 75-µV limit, and you can see the transmitted eye movements in EEG 5 and EEG 6. The EMG is very low, and small rolling eye movements are seen in the EOG.

The δ-frequency (0–4 Hz) dominated in foals even in light sleep. Small rolling eye movements were visible in the EOG. The EMG was characterized by a changing tone and was in comparison with the wakefulness state in the low-tensioned range. The average maximum amplitude height was 87.4 µV (± 24.7), with the maximum amplitude being 188 µV (Fig. 2). In light sleep, the EEG waves were predominantly within the 75 µV limit. Grapho-elements showed spindle-like structures and K-complexes. Many transmitted eye movements were recognized in the EEG waves of electrodes EEG 5 and EEG 6. When light sleep occurred during standing, the foals stood on both front feet and one hind foot, while the other hind foot was rested. The head was lowered, and the ears were held relaxed to the side. In sternal recumbency, the chin was resting on the ground, and the ears were held relaxed to the side. In lateral recumbency, light sleep with both drawn-in and stretched-out extremities could be distinguished.

The average duration of a light sleep sequence was 1.61 min (± 1.58; minimum 0.5 min; maximum 18.0 min) during the day and 1.96 min (± 2.05; minimum 0.5 min; maximum 23.0 min) at night, being significantly (t-test for dependent variables, t = 5.781, *p* < 0.001) longer at night than during the day (Fig. 1A). With 34.2% on average, the foals spent most time in light sleep between midnight and 3.00 a.m. (Fig. 1B). During the night, the foals spent more time in light sleep than during the day (t-Test, t = 7.608, *p* < 0.001).

### Slow-wave sleep

**Figure 3:**
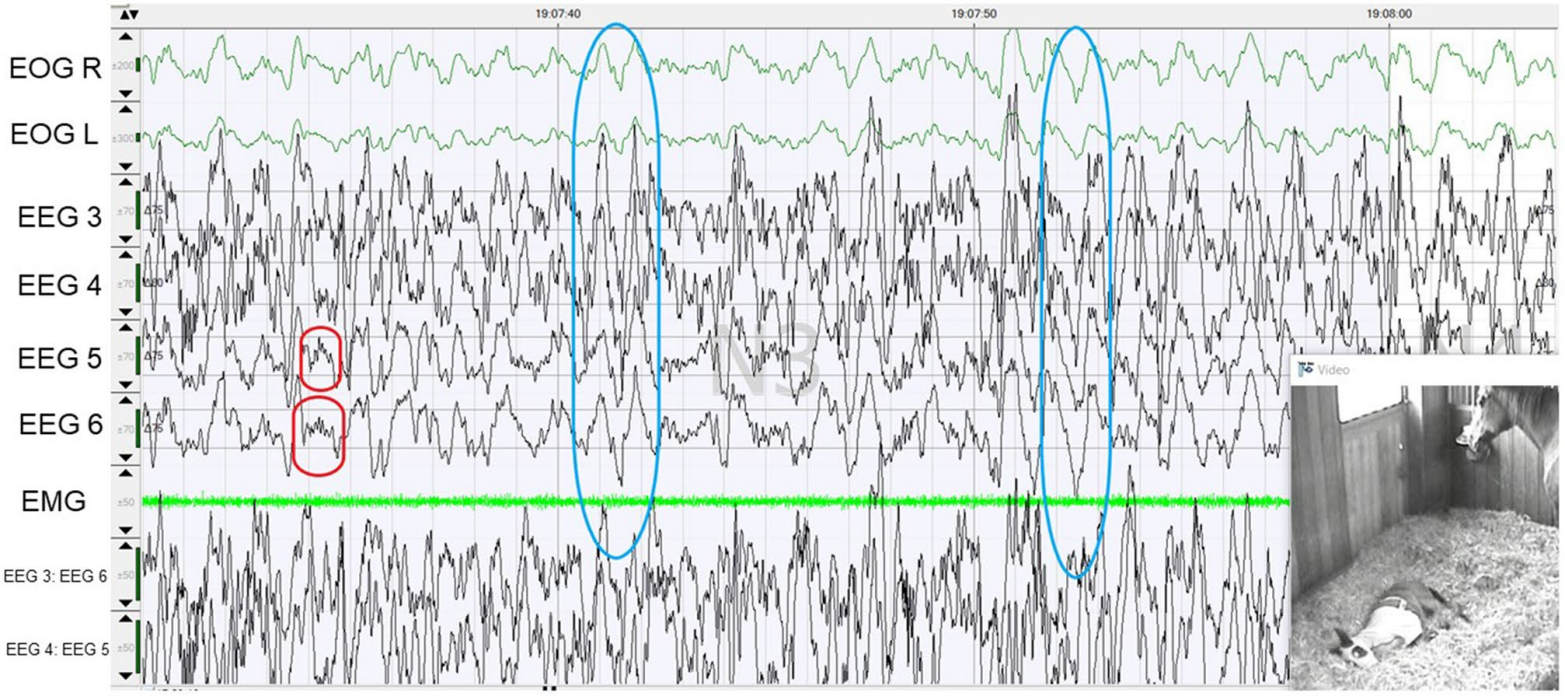
Slow-wave sleep of a foal lying in lateral recumbency. The red circles show the spindles, the blue circles present large amplitudes across all EEG and EOG recordings.

The EEG of slow-wave sleep was characterized by very high amplitudes. The average maximum amplitudes were 281.6 µV (± 96.8), with amplitudes of 586 µV also occurring. The dominant frequencies with approximately 50% were δ-waves (0–4 Hz). We noticed that EEG 3 and EEG 4 had on average fewer high amplitudes than EEG 5 and EEG 6. The EOG reflected the EEG waves, thus not allowing the evaluation of the real eye movements (Fig. 3). Repeated large amplitudes across all EEG and EOG channels were recognizable in all foals (Fig. 3, blue circles). The EMG amplitudes were slightly higher during slow-wave sleep than during REM sleep, but very even. The highest proportion (33.8%) of time spent in slow-wave sleep was observed between 9.00 pm and midnight (Fig. 1B). The average duration of a slow-wave sleep sequence with 5.78 min (± 5.6) in this three-hour period was also the longest (Fig. 1A). The foals spent more time in slow-wave sleep at night than during the day (t-Test, t = − 7.608, *p* < 0.001), and the average duration of the separate slow-wave sleep sequences was also significantly longer at night than during the day (t-test for dependent variables, t = 3.915, *p* < 0.004).

### REM sleep

**Figure 4:**
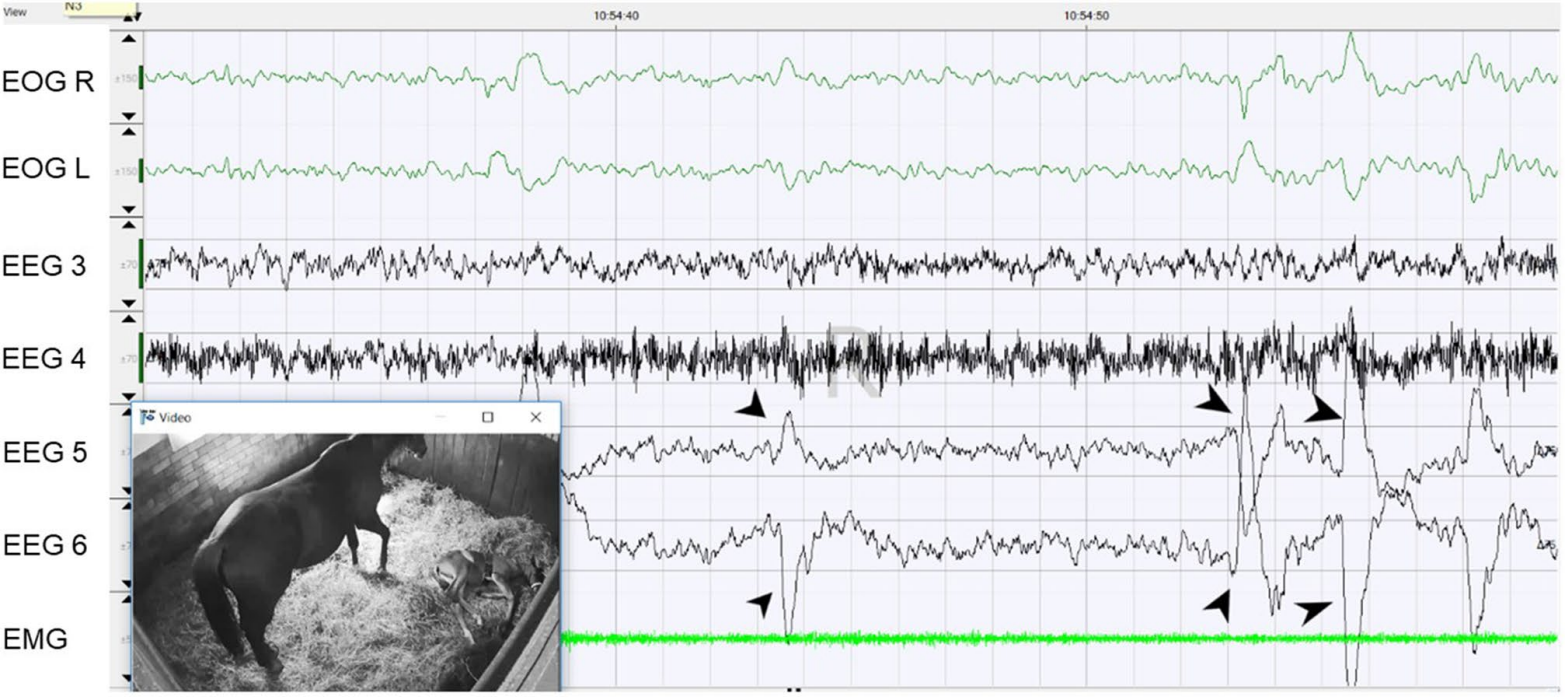
REM sleep of a foal lying in lateral recumbency. The eye movements are transmitted to EEG 5 and EEG 6 (arrowheads).

REM sleep in the EOG of the foals was characterized by rapid eye movements. The EMG showed the least tense state of all stages of vigilance (Fig. 4). No prolonged REM sleep was observed in any of the foals. REM-sleep-like brain waves could be recorded in wakefulness during faeces and urine discharge. Therefore, 57.4% of these REM-sleep-like brain waves were recorded while the foals were standing, whereas 34.4% occurred in lateral recumbency and 7.7% in sternal recumbency. Rapid eye movements were also observed during the transition from wakefulness to a sleep state. The differentiation of rapid eye movements from actual REM sleep was possible by the clearly higher muscle tone shown in the EMG. Considering all vigilance stages, the muscle tone was the lowest during actual REM sleep. During the day, REM sleep could not be observed in every foal. The average percentage of REM sleep over the whole measurement time was lowest (ANOVA, F = 271.96, *p* < 0.001) and the duration shortest (t-test, t = − 12.150, *p* < 0.001) as compared with the other stages of vigilance (Table 1). At night, the foals spent more time in REM sleep than during the day (t-test, t = 2.243, *p* = 0.028) (Fig. 1A and B).

### Lying behaviour

**Figure 5:**
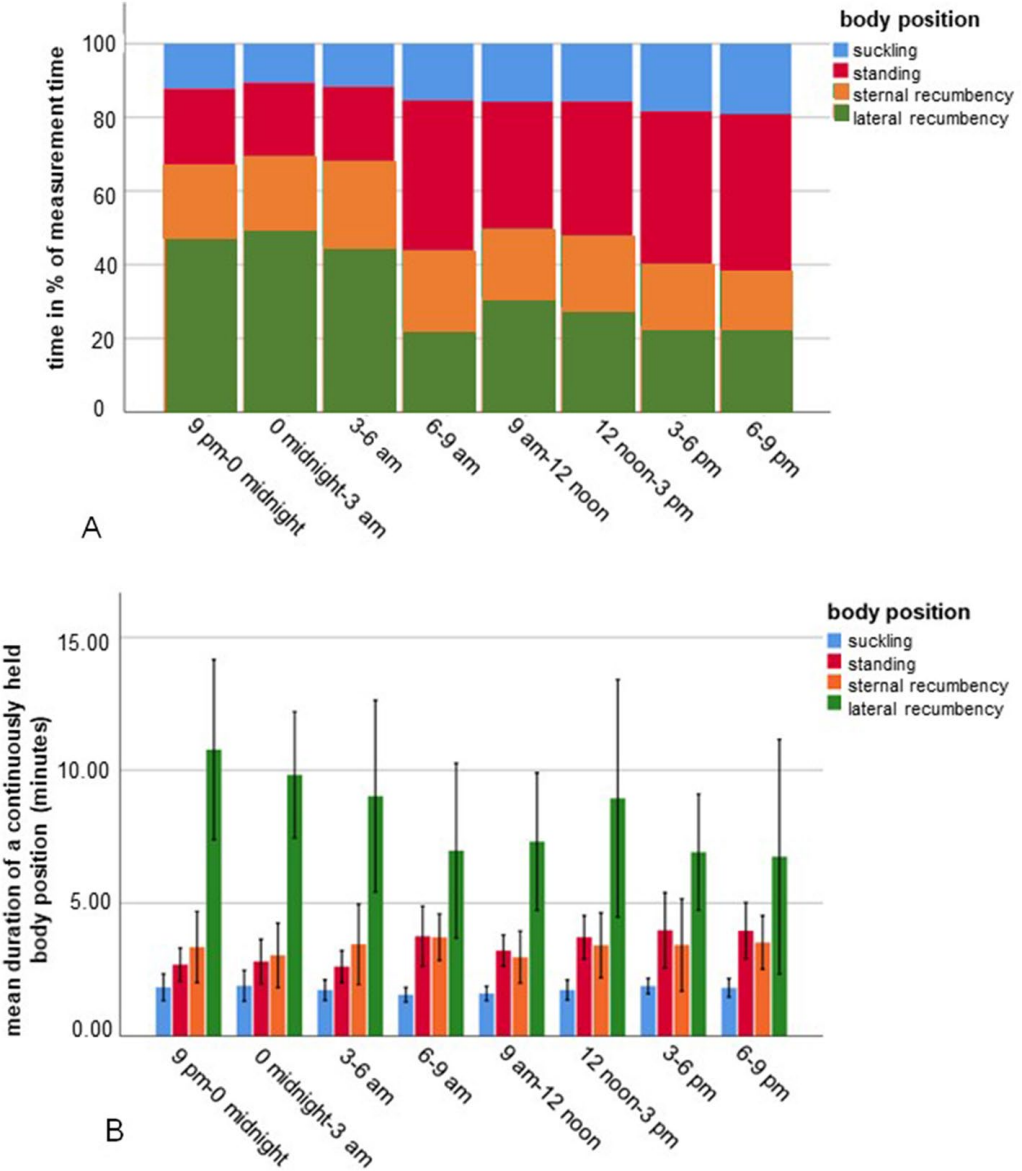
Percentage (**A**) and duration (**B**) of the four body positions of foals (n = 10) during the three-hour periods of a one-day cycle (total measurement time: 48 h). The x-axis shows the three-hour periods of the 24-h cycle. The y-axis presents the percentage (**A**) or the sequence duration in minutes (**B**) of the different body positions. A sequence represents a single continuously held body position. The body positions are presented with different colours (blue: suckling; red: standing; orange: sternal recumbency; green: lateral recumbency).

To analyse the activity and lying behaviour of the foals, we differentiated between standing, suckling, sternal recumbency and lateral recumbency. The percentage of the respective body positions during the total measurement time, the average duration of the separate sequences and the percentage of the body positions in relation to the sleep stages are shown in Table 2 and Fig. 5A,B.

**Table 2:** Overview of the percentage of the total measurement time (48 h) and the duration of the four body positions observed in foals (n = 10) and the percentage of each body position in the four vigilance stages.

Parameter	Body position			
	Standing	Suckling	Sternal recumbency	Lateral recumbency
% of measurement time	32.1	14.8	19.4	33.7
Average duration (± standard deviation) of a sequence^1^ in minutes	3.4 (± 3.7) Minimum 0.5 Maximum 42.5	1.8 (± 0.9) Minimum 0.5 Maximum 10.0	3.2 (± 3.6) Minimum 0.5 Maximum 28.5	8.3 (± 9.8) Minimum 0.5 Maximum 53.5
% in wakefulness	84.6	56.7	49.4	28.5
% in light sleep	6.5	43.2	34.1	44.0
% in slow-wave sleep	1.8	0	15.5	24.0
% in REM sleep	7.1	0	1.1	3.5

^1^Sequence = A single continuously held body position.

### Standing

The foals spent more time standing during the day than at night (t-test, t = 9.657, *p* < 0.001). Most of the time spent standing, with 40.9% of the measurement time, was between 6.00 and 9.00 a.m. The least time spent standing was between midnight and 3.00 a.m. with 21.0% on average (Fig. 5A). REM-sleep-like EEG waves could be observed (during the day and at night) while the foals were releasing urine and faeces, explaining the 7.1% REM sleep during standing (Table 2). The mean percentage of time spent standing increased significantly (spearman´s rho = 0.692, *p* < 0.001) from the beginning of the night until the evening of the next day, with a higher percentage of time spent standing between 6.00 and 9.00 a.m. than in the following three-hour period (t-test, t = − 5.302, *p* < 0.001).

### Suckling

The duration of the suckling sequences between midnight and 3.00 a.m. was the longest with an average of 1.94 min (± 0.97; minimum 0.5 min; maximum 7.0 min), and that of the suckling sequences between 6.00 and 9.00 a.m. was the shortest with an average of 1.57 min (± 0.72; minimum 0.5 min; maximum 4.0 min) (Fig. 5B). The total percentage of time spent suckling increased significantly (spearman´s rho = 0.422, *p* < 0.001) throughout the day until it reached its maximum of 17.8% between 6.00 and 9.00 p.m. (Fig. 5A). The average time spent suckling was longer during the day than at night (t-test, t = 3.827, *p* < 0.001).

### Sternal recumbency

The forelegs of the foals were tucked up in front of or under the breast or stretched out to the front. The hind legs were pulled under the body or stretched out to one side. The head was either set down with the chin on the ground, lay flat on its side, or was carried freely. Between day and night, no difference in the percentage of time spent in sternal recumbency could be observed (t-test, t = − 1.377, *p* = 0.173). However, the probability that the foals spent time in sternal recumbency decreased from night to day to evening (spearman´s rho = 0.277, *p* = 0.043).

### Lateral recumbency

The foals were lying flat on their side, the legs were stretched away from the body, and the head and neck lay flat on the floor. The duration of the respective lateral recumbency sequences was longer than the duration of sequences in other body positions (t-test, t = 11.629, *p* < 0.001). The longest duration of lateral recumbency sequences was between 9.00 p.m. and midnight with an average of 10.59 min (± 11.47; minimum 0.5 min; maximum 53.5 min) (Fig. 5B). At night, the foals spent more time in lateral recumbency than during the day (t-test, t = − 7.605, *p* < 0.001). We observed a significant (spearman´s rho = − 0.562, *p* < 0.001) decrease in the mean percentage of time spent in lateral recumbency from night to day to evening (Fig. 5A). The time between 6.00 and 9.00 a.m. is an exception in the course of the day, because the foals spent less time in lateral recumbency than in the previous three-hour period (t-test, t = 3.912, *p* < 0.001).

## Discussion

According to the American Academy of Sleep Medicine^33^, the vigilance stages in human new-borns under two months of age are classified as awake, NREM sleep (here: non-REM sleep), REM sleep and transitional stages. In the adult horse, the stages were classified as awake, light sleep, slow-wave sleep and REM sleep^7–9^. The classification of sleep stages in foals needs further discussion based on previous studies and the results of this study. Mysinger et al.^16^ found slow frequencies (2–6 Hz) with medium to high voltages (20–90 µV) superimposed by faster frequencies (20–30 Hz) with low to medium voltages (5–25 µV) in the EEGs of alert new-born foals. The EEGs of foals in a relaxed state showed irregular waveforms with slow to medium frequencies (1–6 Hz and 8–10 Hz) and medium to high voltages (10–60 µV to 50–200 µV), and those of foals sedated with xylazine showed slow-wave sleep (1–3 Hz) with high voltages (10–200 µV)^16^. In previous studies on cranial disorders, high-voltage slow waves in adult horses were often described in connection with pathological conditions^23^ or observed after the administration of xylazine, ketamine or isoflurane^15,23,34^.

In the present study, these high-voltage slow waves were classified as slow-wave sleep and considered physiological. They were found in all foals, but none of the foals was neurologically conspicuous, neither during the previous examination nor during the occurrence of high-voltage slow waves. All 30-s intervals in which more than 20% of the waves lie in a range of 1–2 Hz and > 75 µV are defined as slow-wave sleep^35^. It is questionable whether the term ‘slow-wave sleep’ with these limit values is meaningful in foals because both the light sleep and the deep sleep sequences were partly slow-wave sleep. An alternative would be to adjust the limit values for the evaluation of foal sleep. In light sleep, the amplitudes of the waves sometimes exceeded the 75-µV limit by more than 20%, but morphologically the waves can clearly be assigned to the light sleep sequence. By raising the slow-wave sleep threshold to 85 µV, the percentage of light sleep waves exceeding the newly set threshold would be below 20%, which would rule out an assignment to slow-wave sleep.

As with adult horses^6,8,9^, we found no constant α-rhythm in the foals, which in human medicine is a robust indication of relaxed wakefulness. However, in foals in all sleep stages, an α-rhythm was recognizable in the background activity as previously described in adult horses^36^. No 6-Hz spike-wave discharges or bursts of central or parietal β-waves, as described for foals suffering from epilepsy^26^, could be observed among the occurring grapho-elements.

Contrary to the observations of Aleman et al.^36^, the present study did not reveal any prolonged REM sleep in foals. We measured short sequences of REM sleep lasting 3.5 min at most. Previous statements that foals spend 15% of a 24-h period in REM sleep could not be confirmed in this work (the foals spent on average 1.1% of the total measurement time in REM sleep)^37^. One explanation for the varying results could be that in previous studies, a correlation between lateral position and REM sleep was assumed, and the results were not confirmed by polysomnographic examination. We furthermore found that the average percentage of wakefulness during a 24-h period reached its minimum (32.8%) between 9.00 p.m. and midnight. From midnight on, it increased continuously throughout the day until it reached 55.7% between 6.00 and 9.00 p.m. The only exception in the course of the day with 63.3% was between 6.00 and 9.00 a.m. The question arises whether the sleep–wake behaviour of the foals underlies a circadian rhythm or whether the natural sleep behaviour at this time of day was disturbed by the stable operation. Finally, whereas Crowell-Davis^31^ observed during the first week of the foal’s life that 3.6% of sleep occurred in a standing position, we found this percentage to be 8.4% (4.6% of light sleep and 3.8% of slow-wave sleep).

Tateo et al.^38^ observed that foals were standing for 174 min (12.1%) during a 24-h observation period. In the present study, the average proportion of standing in foals was distinctly higher at 32.1%. Tateo et al.^38^ gave an average of three minutes for the duration of the individual standing sequences, which corresponds to our results of 3.4 min. In earlier studies, the relative time spent in lateral recumbency was 15% in new-born foals^29,39^. Murase et al.^32^ observed that 44.6% of the total measurement time was spent in recumbency, which is clearly below the 53.1% observed here. A possible explanation could be that the foals spent more time on the pasture in the study of Murase et al.^32^. Crowell-Davis^31^ observed that, as in the present study, the foals took all four lying positions (sternal and lateral recumbency, each left and right). In that study, recumbency accounted for 31.8% of the total measurement time, and the duration of the individual lying sequences varied but was often longer than 15 min^31^. In the present study, the foals spent on average 53.1% of the total measurement time in recumbency, and the average duration was 8.3 min (± 9.8) in lateral and 3.2 min (± 3.6) in sternal recumbency. The different results can potentially be explained by different observation methods. Because Crowell-Davis^31^ used several 15-min observation periods for evaluation, whereas we ensured continuous video surveillance, we assume that the higher percentage of time spent in recumbency is more realistic. In addition, Crowell-Davis^31^ observed the foals in the herd on the paddock whereas we observed each foal in the box only in the presence of its mother.

The significantly higher proportion of standing sequences and the lower proportion of time spent in lateral recumbency between 6.00 and 9.00 a.m. when compared with the previous three-hour period is striking. This result was accompanied by a significantly higher average percentage of wakefulness and a lower percentage of slow-wave sleep*.* Thus, we assume that the foals were disturbed by stable work during the period from 6.00 to 9.00 a.m. and their normal sleep and lying behaviour patterns were interrupted. On average, the most slow-wave sleep with 33.8% and the least wakefulness with 32.8% occurred in the period between 9.00 p.m. and midnight. Based on these observations, we recommend that work in the foal stable should be postponed to the late afternoon, because the foals are most awake in the time from 3.00 to 9.00 p.m., with > 40% of standing and > 54% of wakefulness recorded then. Furthermore, the stable should be quiet from 9.00 p.m. at the latest, because between 9.00 p.m. and midnight is the most important time for the foals to sleep.

The foals were observed suckling during all periods of the 24-h course. The average percentage of suckling time was significantly higher during the day than at night. The duration of the suckling sequences did not differ between day and night. The length of the suckling act varies greatly in the available literature. It ranges from 1.04 ± 0.05 min^40^, 86.4 ± 6.1 s^30^, 147 s^41^, to a maximum of 198 (73.8 ± 28.9) seconds^42^. Our result of 1.8 ± 0.9 min is in the midrange compared with the other studies. One explanation could be that the foals in our study were able to drink undisturbed by other horses and were not distracted by human observers, because we used video cameras. The average percentage of drinking sequences in our study was 14.8%, which is slightly higher than in Tateo et al.^38^ with 12.2%, where similar external circumstances prevailed.

Our method of electrode application proved to be suitable for polysomnographic studies on young foals. Nonetheless, we recommend a few adjustments (especially in the placement of two EEG electrodes, see below) for improvement in further studies. In principle, it was possible to record high-quality EEGs, EMGs and EOGs over an extended period, as found by other authors^7,8,43^. In contrast to the observations of Lacombe et al.^23^, it was possible to apply the electrodes without sedation or general anaesthesia, and the results were easy to evaluate. As also described in previous studies, the evaluation of the recordings is always influenced by the subjective assessment of the evaluator, because there is still no system for the computer-assisted evaluation of horse or foal sleep^23^. In human medicine, polysomnographic data are now analysed by machine. Only ambiguous areas need to be evaluated by humans. This saves a substantial amount of time and results in standardized data. The results of this study should serve as a guideline for further analyses of sleep in foals.

The application of the electrodes and the polysomnograph took about 15 min in the present study. The foals were habituated to human handling, so sedation was not necessary. They were only fixed in the foal grip. Because sedatives and narcotics have an influence on the brain waves of adult horses and foals^16,23,34^, it was important to avoid them to observe the physiological brain waves of young foals. As described by Toth et al.^44^, foals relax when they are held by humans (body temperature decreases, heart rate decreases); we could not observe the described EEG changes (spindles, vertex-peaks, K-complexes and slow waves) during application of the electrodes.

Only a combination of superglue and electrode conductive paste could ensure that the electrodes remained fixed over a longer period. Removing the electrodes after 48 h was no problem, but the mix of superglue and electrode conductive paste still adhered to the foal’s skin for several days to weeks. Skin irritations were not observed, but the shorn areas were still marked months later by different coat colours, which was inconvenient for the owners. However, no lasting damage was observed. An alternative skin adhesive would be collodion, which was also used in previous studies^36^. Cousillas et al.^45^ used a halter-like headset in which the electrodes were integrated. The application time was less than five minutes, and there was no need to shave the skin or use superglue^45^. However, measurements were only carried out on awake adult horses and only for a period of 15 min each. Furthermore, EOG and EMG recordings were not possible with this system, which is why it does not seem useful for determining the sleep stages of foals. Wijnberg et al.^43^ in their study of adult horses initially used cup electrodes, which they glued onto previously shaved and alcohol-cleaned skin with collodion. After the first five horses in the study, the authors switched to self-adhesive electrodes because they were easier to apply and safer for the horses^43^.

In the study by Wijnberg et al.^43^, it was possible to insert the electrodes individually into the headbox of the polysomnograph. This was not possible with our system because all 10 electrodes were connected in a common wiring harness. This system eliminated the possibility of allowing the foals to pause during the application of the electrodes. We placed the electrodes according to the same scheme as used on adult horses^7,16^, which is also recommended by the American Academy of Sleep Medicine for humans^46^. A disadvantage of this placement was the transfer of eye movements to the electrode pair EEG 5 and EEG 6. The most likely cause is that the electrodes were placed near the nasal canthus, whereby the eye movements were recorded. For future recordings, it would therefore be useful to place the electrode pair EEG 5 and EEG 6 further occipitally to reduce the artefacts by eye movements.

As soon as the electrodes no longer had optimal coupling or were glued to a muscle, the EEG recordings were superimposed by artefacts, which was also described in previous studies^9,47^. The coupling of the electrodes became notably worse, and the electrodes loosened more often the longer they were on the foal. Lacombe et al.^23^ injected muscle groups with a local anaesthetic to avoid artefacts caused by muscles. This was not an option in the present study because the local anaesthetic would have to be placed under general anaesthesia and no local anaesthetic was available that would have worked for 48 h^48^. Moreover, local anaesthesia does not seem practical for future studies.

Due to the relatively large number of artefacts, such as sweat and movement artefacts, and the detachment of individual electrodes, a quantitative computer-aided EEG evaluation of our data is not meaningful. There is currently no alternative to manual evaluation of the measurement, which is also recommended by other authors^34,49^. The values for the EEG waves presented in the results section come from artefact-free sequences of the measurements. Morphologically, sequences with artefacts can also be evaluated and clearly be assigned to the different sleep stages. However, it is difficult to classify these into fixed values, which is why a manual evaluation of the respective measurements is necessary. A disturbance of the horses by the mounting of the cameras could not be avoided because the video recordings were required for the evaluation of the recorded material^9,36^.

Our results demonstrate that the EEGs of foals under one week of age differ noticeably in their morphology from the EEGs of adult horses. The results and procedures presented here will serve as a guideline for the evaluation of brain waves of young foals in the future as well as for recommendations for animal-friendly keeping of foals. Based on the results, we recommend stall rest from 9.00 p.m. at the latest, because the most important sleep period of the foals is between 9.00 p.m. and midnight. Furthermore, stable work should be postponed to the late afternoon because the foals sleep the least during this period. Especially for hand-fed foals, it is important to note that foals under one week of age suckle during all periods of the 24-h course, with more suckling activity during the day than at night.

## Animals, materials and methods

### Animals

Ten healthy foals were measured in their domestic stables to record their natural sleeping, lying and suckling behaviour polysomnographically. To be included in the study, the foals had to be clinically healthy during the previous examination, the gestation period had to have lasted between 320 and 360 days, and the foals had to spend at least the nights alone with their mother in a stall that was completely captured with video cameras. The box size of all measured foals was above the minimum required, i.e. ≥ (2.3 × withers height)^2^, for mares with foals as stated by the Federal Ministry of Food, Agriculture and Consumer Protection (Bundesministerium für Ernährung, Landwirtschaft und Verbraucherschutz, BMELV) in the guidelines for the assessment of horse husbandry under animal welfare aspects^50^. In addition, the measurements had to take place in the first week of the foal’s life. During the day, it was up to the owners how much time the foals could spend on the pasture in order not to change the daily routine of the foals. The foals were let out of their boxes for only short periods (maximal 30 min). During this time, monitoring was performed exclusively with the polysomnograph and not additionally with video cameras. Both the polysomnographic measurements and the additional video recordings were made for each foal throughout 48 h. Before the examination of the foals was conducted, the owners had to sign a consent form.

### Polysomnograph

The polysomnograph used for the measurement was the portable SOMNOscreen-plus from SOMNOmedics GmbH (D-97236 Randersacker). Including the battery, the weight of the device was 220 g with a dimension of 140 × 70 × 22 mm. The polysomnograph was attached with a belt to the distal part of the foal’s neck. The data were stored simultaneously on a 2-GB high-speed compact flash card in the polysomnograph and on the hard disk of a Dell notebook. Data transfer to the notebook, which was placed outside the horse box, occurred with 16-bit resolution via a radio module attached to the SOMNOscreen-plus.

### Electrode placement

**Figure 6:**
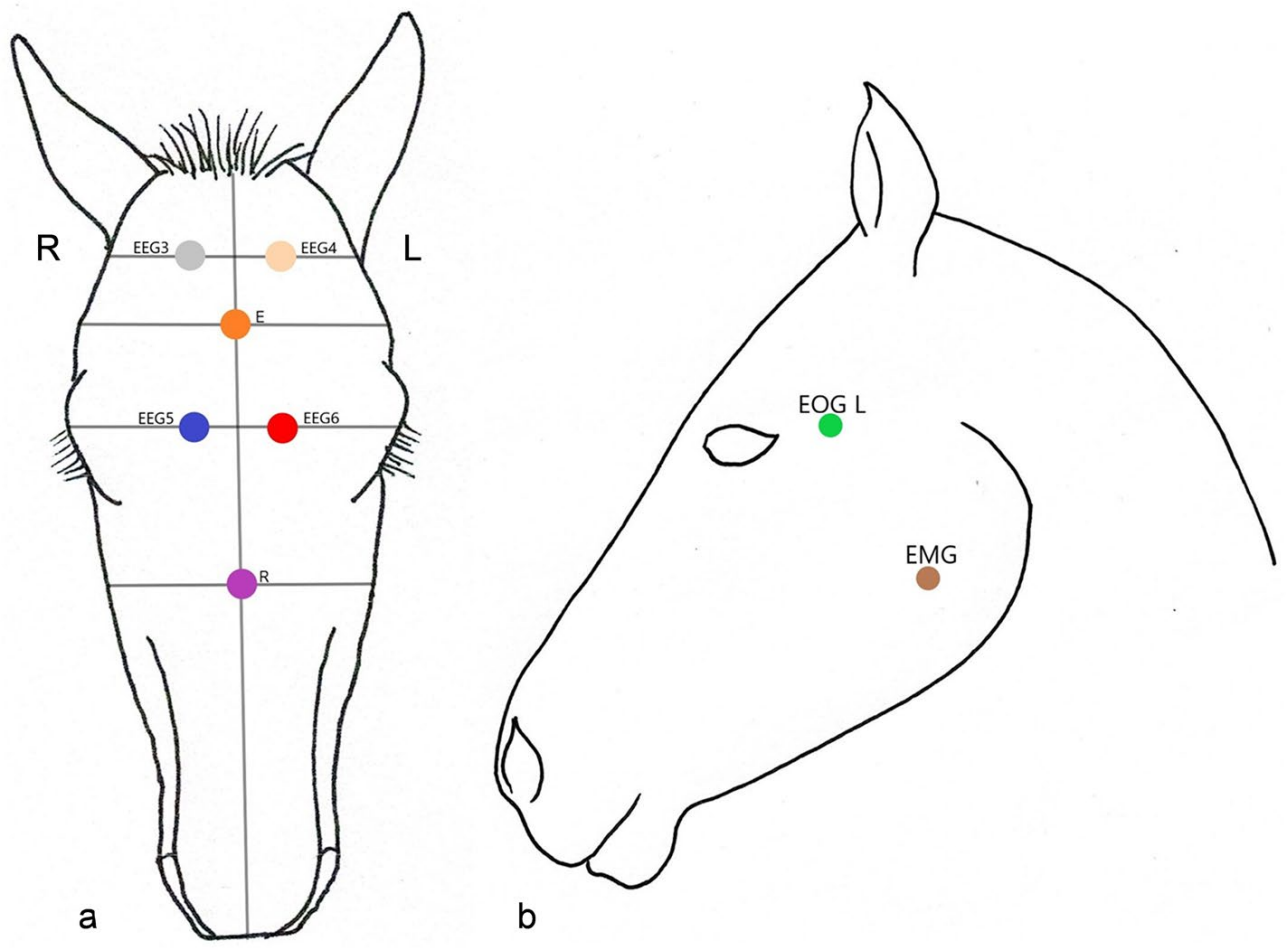
Electrode placement: (**a**) EEG 3 and EEG 4: occipital; EEG 5 and EEG 6: frontopolar; E and R: reference electrodes; (**b**) EOG and EMG shown on the left side of the head (modified according to Güntner^7^ and Williams et al.^9^).

The electrodes used were 10 gold-plated cup electrodes, which were one-meter-long, differently coloured cables that ran together in a common cable harness, which was connected to the polysomnograph. The cup electrodes were filled with EC2 Genuine Grass Electrode Cream, which is a special conductive adhesive paste for cup electrodes, and their rim and stem were super coated with UHU all-purpose adhesive. This combination enabled the electrodes to be securely attached on the foal’s head. For additional fixation and protection, the electrodes were covered with Snøgg Animal Polster, which is a self-adhesive foam. The skin areas on which the electrodes were glued were sheared, degreased with isopropyl alcohol and cleaned. The electrodes were applied to each foal’s head according to the application scheme of Güntner^7^ and Williams et al.^9^ (Fig. 6). The two occipital electrodes (EEG 3 and EEG 4) were placed each 1.5 cm left or right of the midline of the os frontale, at the level of the ear base. The two frontopolar electrodes (EEG 5 and EEG 6) were placed each 1.5 cm left or right of the midline of the os frontale, at the level of the lateral canthus. The two reference electrodes (E and R) were placed on the midline of the os frontale, the upper one at the level of the cheekbone, the lower one at the level of the middle of the crista facialis. The two EOG electrodes (EOG L and EOG R) were placed 1.5 cm lateral to the temporal canthus on each side. The two EMG electrodes were placed on the musculus mentalis, 1.5 cm orally to dens praemolaris 2 on each side (Fig. 6). The foals were held in the foal grip for application of the electrodes. The person holding the foal gripped the tail with one hand and placed the other hand under the head of the foal. If necessary, an ear could be grasped with the front hand.

### Video camera

In addition to the infrared camera belonging to the system (video camera COMPACT, SOMNOmedics), which films synchronized with the discharges of the polysomnograph, one to four further infrared cameras (CCD CAMERA) were installed depending on the spatial conditions of the stables. The latter ensured a continuous recording of the foals.

### Data analyses

The results were analysed manually using the DOMINO analysis software. Each 30-s interval was assigned a stage of vigilance and a body position. If two stages of vigilance occurred in one interval, the stage that prevailed over 50% of the time was selected. Twenty-four hours were divided into eight periods of three hours each. Additionally, a subdivision into day (from 6.00 a.m. to 9.00 p.m.) and night (from 9.00 p.m. to 6.00 a.m.) was made. The body positions were divided into standing, suckling, sternal recumbency and lateral recumbency (Table 3). The stages of vigilance were divided into wakefulness, light sleep (which includes stages N1 and N2 of non-REM sleep according to the manual of the American Academy of Sleep Medicine^33^), slow-wave sleep and REM sleep. A single continuous vigilance stage or a single continuously held body position was described as a sequence. For each three-hour observation period, the sequences of each vigilance stage or those of each body position were summarized and presented as percentage of the observation time and as average duration (± standard deviation) in minutes.

**Table 3:** Ethogram describing the behaviour of the foals in the four assessed body positions.

Body position	Description of behaviour
Standing	All behaviours that the foal shows when standing, except suckling. Included are playing, standing, walking, trotting, galloping, exploring the environment, and making contact with other horses
Suckling	As soon as the foal touches the mother's udder with its mouth. Both the actual drinking process and the priming of the udder are included in the term suckling
Sternal recumbency	The foal lies in an upright position on its sternum and abdomen. The front legs are bent under the body or extended forward. The hind legs can be stretched out to the side or are also bent under the body. The head is carried freely or supported with the chin on the ground
Lateral recumbency	The foal lies flat on its side. The front and hind legs are extended or slightly bent next to the body. The head lies flat on the side, resting on the lower half of the face

### Statistical analyses

The statistical analyses were performed using the SPSS statistical software (versions 25.0 and 26.0, IBM Corp.). For nominal data such as body position and sleep stage, the relative frequency in percent of its occurrence was calculated for each individual animal and for each specific three-hour period. Afterwards, the obtained values were analysed statistically for all animals according to metric data. Stacked bar charts were used for the graphical representation of relative frequencies. For metric data, the arithmetic mean ± standard deviation, the median, the minimum and the maximum were calculated as parameters of the distribution. To differentiate between normally distributed and non-normally distributed data, the Shapiro–Wilk test was performed subsequently. All metric data revealed normal distributed. For the graphical representation of normally distributed data, bar charts were applied. The Spearman correlation coefficient *rho* was calculated to quantify correlations between metric variables and the period. Mean differences between two normally distributed metric variables were tested for hyper randomness by using the *t-*test or, for dependent samples, the pair comparison test (i.e. the *t*-test for dependent variables). In cases with more than two characteristics for the grouping variable, the single-factor analysis of variance (ANOVA) with Bonferroni post-hoc tests was applied. The selected significance level was *p* < 0.05.

### Ethical statement

The project, including the study design, was approved by the institutional animal research ethical committee of the Ludwig-Maximilians-University Munich (protocol number 106-15-01-2018). All tests on the foals were carried out in compliance with the latest guidelines and regulations. Additionally, the owners of the foals had to sign a declaration of consent.

## Data Availability

The datasets generated and analysed during the current study are available from the corresponding author.
